# Molecular phenotyping of plant single cell-types enhances forward genetic analyses

**DOI:** 10.3389/fpls.2015.00509

**Published:** 2015-07-06

**Authors:** John Schiefelbein

**Affiliations:** Department of Molecular, Cellular, and Developmental Biology, University of Michigan, Ann Arbor, MI, USA

**Keywords:** genetic analysis, mutant, molecular phenotype, cell biology, transcriptomics

## Abstract

Recent advances in the isolation of single cell-types in plants provides an opportunity to conduct detailed analyses of their molecular characteristics at high resolution. This kind of cell-type specific molecular phenotyping is likely to enhance forward genetics studies to dissect the effect of mutations and thereby aid gene function assignment. Recent experimental results support this view, demonstrating that different cell-types exhibit substantial variation in transcript, protein, and metabolite accumulation and these molecular phenotypes are often sensitive to genetic and environmental alterations. The use of single cell-type molecular phenotyping approach to define plant gene function is most amenable to cell-types with well-characterized molecular tools and isolation protocols.

## Introduction

The use of forward genetic analysis has historically been an effective approach for defining gene function. By analyzing the phenotypic effect of a mutated gene, it has been possible to assign hundreds of genes in numerous organisms to particular developmental or metabolic pathways. However, a limitation of this approach is its reliance on the detection and measurement of phenotypic alterations. Indeed, if one cannot observe a change in the phenotype of a genetically altered individual, then little insight is gained concerning the affected gene’s role in the process of interest.

The recent development of approaches to isolate and analyze plant single cell-types are likely to help overcome this limitation of forward genetics in plant biology. Specifically, recent studies suggest that individual plant cell-types exhibit distinct molecular characteristics (molecular phenotypes) and that these are differentially affected by genetic or environmental changes. Thus, forward genetic analyses that focus on phenotyping single cell-types are expected to have the capacity to detect and measure molecular alterations and thereby provide greater insight into gene function.

This perspective article summarizes recent studies showing distinct molecular phenotypes in plant single cell-types, their sensitivity to genetic and environmental perturbation, and evidence that forward genetic analyses are aided by single cell-type studies. This article also examines the factors likely to be critical for the successful use of molecular phenotyping to study gene function at the level of plant single cell-types.

## Molecular Phenotypes in Single Cell-Types of Plants

The molecular analysis of a bulk cell population yields the composite molecular phenotype of many cell and tissue types, leading to an average assessment of the transcriptome, proteome, or metabolome of the population. Although such bulk data are useful for some purposes, recent studies have shown that individual cell-types within a plant organ possess widely disparate molecular characteristics (Brandt, 2005). For example, extensive microarray analyses of cell-sorted *Arabidopsis* roots show cell-type specific transcript accumulation that differs substantially from the total root RNA population (Birnbaum et al., 2003; Brady et al., 2007). Similar cell-specific expression has been observed in several different isolated cell-types from rice, yielding a “cell-type transcriptome atlas” (Jiao et al., 2009). Further, it has been shown that distinct protein and metabolite accumulation patterns exist in different root cell populations as compared with the entire root organ (Petricka et al., 2012; Moussaieff et al., 2013). Although the evidence for cell-type specific accumulation of biological molecules is greatest for roots, the analysis of other isolated cell-types of plants, including trichomes and stomata, also reveals transcriptome patterns distinct from the larger organ-level accumulation patterns (Lieckfeldt et al., 2008; Adrian et al., 2015).

Furthermore, it is now clear that the molecular phenotype of a given cell-type is altered in a unique manner following genetic or environmental perturbation. That is, individual cell-types appear to respond in different ways to a particular alteration, such as a mutation or an environmental stress. For example, the analysis of single-gene mutants shows distinct transcriptome responses at the cell-type level in roots (Brady et al., 2011; Bruex et al., 2012), cotton fibers (Wan et al., 2014), and trichomes (Jakoby et al., 2008). Further, distinct cell-type-specific alterations in transcriptional profiles are observed following a specific change in the environmental conditions, including iron deprivation, nitrogen availability, or salt stress, that likely reflect the physiological response appropriate for a particular cell-type (Dinneny et al., 2008; Gifford et al., 2008; Geng et al., 2013). These results indicate that single cell-types of plants have distinct molecular programs that are not apparent from the analysis of whole organs or whole plants.

An important implication of these studies is that high resolution molecular analyses at the single cell level may be useful to improve forward genetic analyses. That is, although a given mutant individual may not exhibit an observable alteration at the level of the whole plant or from a bulk cell population, it may be possible to detect a molecular phenotype if single cell-type analyses were performed. Indeed, the results of several recent studies support this view. First, the molecular analysis of different wild-type *Arabidopsis* lines (ecotypes) has demonstrated substantial differences in their transcript, protein, and metabolite profiles (Keurentjes et al., 2006; Kliebenstein et al., 2006; Fu et al., 2009; Terpstra et al., 2010), indicating a surprising degree of underlying molecular variation that does not cause observable morphological differences, perhaps due to “phenotypic buffering.” Interestingly, these studies also enable the identification of new kinds of quantitative trait loci (QTLs) that likely mediate the responses of large numbers of genes for ecotype-specific traits. Further, the reverse genetic analysis of a collection of stele-enriched *Arabidopsis* transcription factor genes showed that, among the resulting single-gene mutants, 65% of them exhibited reproducible transcriptome changes whereas only 16% exhibited observable morphological changes in the root (Brady et al., 2011). Together, these studies suggest that a substantial degree of molecular variation exists that does not impact the plant’s phenotype.

Finally, direct demonstration of the value of single cell-type analyses for forward genetic analyses has recently been reported. Several studies have shown that a comparative transcriptome analysis of single cell-types from mutants versus wild-type provides enhanced resolution for transcript changes. Comparing cotton fiber transcriptomes from lines differing in fiber production, specific transcription factors and metabolic pathways were identified as fiber associated (Wan et al., 2014). Further, transcript profiles from trichomes of a wild-type and immature trichome mutant lines uncovered new genes required for normal trichome formation (Marks et al., 2009). In another study, several single-gene mutants associated with *Arabidopsis* root epidermis development, but lacking an observable morphological phenotype due to redundancy, were found to alter root epidermis transcript profiles in a manner that reflects the known biological role of the genes (Simon et al., 2013). In addition, the function of an uncharacterized gene in this root epidermal network (*TTG2*) was deduced by inspection of the cell-specific transcriptome of its corresponding mutant, which lacked a morphological abnormality (Simon et al., 2013). Interestingly, the transcript changes showed that TTG2 normally promotes root hair cell differentiation, rather than non-hair cell differentiation as previously suspected. This shows that the high resolution analysis of single cell-types can reveal molecular differences associated with gene function.

## Issues to Consider for Molecular Phenotyping of Single Cell-Types

The studies described above suggests it is possible to better understand gene function by conducting detailed molecular phenotyping of genetically altered lines (e.g., mutant lines). In particular, it is notable that even mutants lacking an outward (morphological) phenotype may exhibit a molecular phenotype at the cell-type level. This is exciting because the majority of single-gene knockouts in plants lack observable changes (Pickett and Meeks-Wagner, 1995; Bouche and Bouchez, 2001; Hanada et al., 2009; Perez-Perez et al., 2009; Lloyd and Meinke, 2012), and so this approach may enable their associated genes to be assigned a particular function.

For the successful application of this approach, there are several issues that should be considered. First, the biological material to be analyzed should be as specific as possible. Ideally, a single cell-type or cell state (specific stage of a given cell-type) should be analyzed. This is important as it is becoming apparent from many different studies that there is tremendous cell–cell variation within multicellular organisms. For example, the oligodendrocyte of the mammalian brain has historically been considered to be a single cell-type, but it is now know to be composed of six distinct subpopulations in the adult mouse brain (Zeisel et al., 2015). One of the limiting factors in isolating single cells is the accessibility of individual cell-types, with epidermal cells (e.g., root hairs, trichomes, cotton fibers) being among the first to be analyzed due to their extension from the plant surface (Qiao and Libault, 2013; Becker et al., 2014). The difficulty in isolating other single cell-types is being mitigated by new advances in protoplasting/fluorescence activated cell sorting (FACS), laser capture microdissection (LCM), or nuclear tagging in specific cell-types (INTACT) that enable cells of internal tissues to be purified in plants (Kerk et al., 2003; Deal and Henikoff, 2010). Another limiting factor can be the small amount of biological material collected, which may prevent accurate assessment of molecule accumulation given current transcriptome, proteome, and metabolome methodologies. Indeed, this may prevent the detection of a “phenotype” in some mutants that elicit relatively minor effects. As the sensitivity of these large-scale methods increases, the ultimate goal is to conduct these molecular analyses on individual cells of a single cell-type. For example, it is conceivable that transcriptomes will soon be possible from single cells through the development of single cell RNA-seq methods (Saliba et al., 2014). It is likely that the combined technical advances in single cell isolation and molecular analysis methods will be required for future success.

Second, the likely success of using this approach is improved if the investigator has some knowledge of the likely defect in the mutant line. This knowledge enables the investigator to focus their molecular analysis on one (or a limited number of) cell-type(s) in the mutant line. In the absence of some knowledge or interest in a particular cell-type(s), it would be difficult to employ this approach to deducing the role of a gene, due to the time and resources required to survey a large number of cell/tissue types and developmental stages of the mutant plant. Having said this, at some point in the future, it may be feasible to consider the ultimate goal of defining the molecular impact of every gene knockout on every cell-type in the plant. It is likely that this information would profoundly change our view of gene activity and function in plants.

Third, this approach is most likely to be successful if there is a known molecular pathway or molecular markers for the cell-type or trait of interest already available to aid in the assignment of gene function. For example, if some of the genes have been identified in a transcriptional regulatory network for the cell-type of interest, then it is a relatively straightforward exercise to determine whether/how the mutant lines alter specific genes or subdomains of the gene network using transcriptomic approaches. This feature has been exploited in the successful applications of this approach to date, using the detailed knowledge available in the trichome and root hair systems (Marks et al., 2009; Simon et al., 2013).

In summary, recent advances in the isolation and characterization of plant single cell-types shows great promise in enhancing the effectiveness of mutant phenotyping. By focusing on single cell-types, rather than entire organs or entire plants, it is possible to detect specific molecular changes in mutant lines. This represents a valuable tool for the future detailed dissection of gene function in plants.

### Conflict of Interest Statement

The author declares that the research was conducted in the absence of any commercial or financial relationships that could be construed as a potential conflict of interest.
